# HOS15 together with PWR–HDA9 positively regulates dark-induced senescence in Arabidopsis

**DOI:** 10.1080/15592324.2025.2564962

**Published:** 2025-09-29

**Authors:** Shah Zareen, Akhtar Ali, Min Jae Bae, Nassem Albakri, Kisuk Park, Hyeseon Yun, Dae-Jin Yun, Junghoon Park

**Affiliations:** a School of Advanced Biotechnology, Institute of Science and Technology, Konkuk University, Seoul, South Korea; b Global Plant Stress Research Center, Konkuk University, Seoul, South Korea

**Keywords:** HOS15–PWR–HDA9, complex, histone modification, senescence, Arabidopsis

## Abstract

Senescence is a conserved phenomenon in all living organisms, including plants. The initiation and progression of leaf senescence can be triggered by natural internal factors or induced by external stress conditions. Over the past few decades, several transcriptional regulators, histone deacetylases/acetyltransferases (HDACs/HATs), signaling transduction pathway components, hormonal regulators, and other proteins have been extensively studied and reported to play a role in regulating leaf senescence. However, a deeper molecular understanding of their mechanisms is needed. We recently reported that a WD40-repeat domain protein, HOS15, regulates aging- and dark-induced senescence. Loss-of-function HOS15 mutant plants exhibited a late senescence phenotype with greater chlorophyll content accumulation. The transcript levels of senescence-related (*SAG12*, *SAG29*, and *ORE1*) genes were downregulated in *hos15-2* plants compared with those in wild-type (WT) plants, whereas photosynthesis-related (*CAB1* and *RBCS1A*) genes were upregulated. Our studies also revealed that HOS15 works together with PWR–HDA9 complex to associate with the promoters and negatively regulates the expression levels of the senescence negative regulators *NPX1, APG9*, and *WRKY57*. Moreover, *hos15-2* plants increased H3 acetylation levels, similar to those of *hda9* and *pwr* plants compared to those of WT plants. In addition, the H3 acetylation level was reduced in the dark-induced senescent leaves in WT plants, but not in the *hos15-2* plants*,* which suggests that dark-reduced H3 acetylation requires functional HOS15. Taken together, we conclude that HOS15 together with the PWR–HDA9 complex epigenetically regulates aging- and dark-induced senescence through a common set of genes in Arabidopsis.

## Background

In plants, senescence is a natural regulatory process that is characterized by declining leaf viability, is often visible through changes in leaf color, and is considered a distinct form of programmed cell death.^1^ Due to the nature of photoautotrophy, plants primarily rely on their leaves to support growth and development. During senescence, plant leaves are no longer photosynthetically efficient, thereby reallocating valuable resources to other organs.^2^ Despite the prominence of senescence processes, the mechanisms that control their initiation, such as signaling, gene expression regulation, nutritional degradation, and remobilization, have been extensively studied.^3^ Abiotic stresses play important roles in plant physiological processes and stress responses as well in determining the progression of leaf senescence.^3^^,^^4^ Similar to other environmental stresses, dark light stress is also essential for plant growth and plays an important role in senescence regulation.^5-7^

 Over the past three decades, age-dependent leaf senescence has been exclusively studied as a process that is fully controlled by genetics.^8^ It is tightly regulated by chromatin dynamics as well as by transcriptional, post-transcriptional, translational, and post-translational modulators.^7^^,^^9^^,^^10^ In addition, several SENESCENCE-ASSOCIATED genes (SAGs), photosynthetic genes, and NAC family transcription factors have been investigated, and their expression is substantially influenced by age-dependent and dark-induced senescence.^11-16^ The transcription factors PHYTOCHROME-INTERACTING FACTORS (PIFs) play a key role in dark-induced senescence.^17^^,^^18^ Furthermore, EARLY FLOWERING 3 (ELF3) and phytochrome B (PhyB) inhibit leaf senescence by suppressing the transcription and protein abundance of PIF4/PIF5. PIF4/PIF5 activates the expression of the ETHYLENE-INSENSITIVE3 (EIN3), ABA-INSENSITIVE 5 (ABI5), and ENHANCED EM LEVEL (EEL) transcription factors.

Epigenetic modifications potentially affect gene transcription, transposon silencing, and genomic stability, as well as the expression of senescence-associated proteins.^19^ DNA methylation, histone modification, chromatin remodeling, RNA methylation, and non-coding RNA regulation provide a set of interrelated pathways that alter the conformation of chromatin to stabilize gene expression in a broad sense.^20^ In histone modifier enzymes, histone acetyltransferase (HATs) and histone deacetylase (HDACs) enzymes are directly linked to gene activation and repression, have been extensively studied in the context of leaf senescence regulation.^21^ However, HDACs are primarily associated with the repression of gene expression.^19^ Similar to other HDACs, the Arabidopsis RPD3-like HISTONE DEACETYLASE 9 (HDA9) regulates the expression of target genes through histone modification, thereby influencing various physiological processes and stress responses.^22^ For instance, HDA9 regulates flowering time, the drought stress response, plant immunity, cell elongation through auxin signaling, the circadian rhythm, the salt stress response, thermogenesis, light-mediated autophagy, the plant vegetative phase, aging- and dark-induced senescence, mRNA biogenesis, heat stress, and seedling traits.^15,23-37^ HDA9 also functions in a complex with other components to repress gene expression.^22^ One such component in Arabidopsis is POWERDRESS (PWR), which encodes a protein containing two SANT domains.^38^ PWR interacts with HDA9 and functions within the same complex to reduce the levels of histone acetylation at the promoter of the target genes, thereby repressing their transcription and contributing to the regulation of various physiological processes and stress responses.^22^ For example, PWR is involved in aging- and dark-induced senescence, floral transition, response to cold and drought stress, thermogenesis, systemic acquired resistance, and seed germination tolerance to high temperature, as well as plant growth and development.^15,25,26,38-43^ These reports highlight the important role of HDA9 and PWR in regulating various physiological processes and stress responses.

HIGH EXPRESSION OF OSMOTICALLY RESPONSIVE GENES 15 (HOS15) is a WD40-repeat domain protein with multifaceted functions that acts as a transcriptional co-repressor.^44-46^ HOS15 is involved in freezing and drought stress responses, flowering time, abscisic acid (ABA)-signaling, plant immune response, leaf senescence, miRNA biogenesis, thermotolerance, and alternative splicing.^26,28,39,44,46-54^ Moreover, the dual functions of HOS15, as an E3 ubiquitin ligase and component of the chromatin remodeling complex, have been exclusively discussed.^22^ Furthermore, HOS15 is a core component of the PWR–HDA9 repressive complex and co-regulates the transcriptional activity of numerous genes involved in plant growth and development.^40^ The loss-of-function HOS15 mutation has been shown to cause hyperacetylation and alteration in methylation, which is similar to observations in *pwr* and *hda9* mutants.^40^ Previously, PWR and HDA9 were shown to be involved in age-dependent and dark-induced senescence through the repression of the expression of the senescence negative regulators *AUTOPHAGY 9 (APG9), WRKY family transcription factor 53 (WRKY53)*, and *NUCLEAR PROTEIN X1 (NPX1).*^15^ We have also recently shown that HOS15 is involved in dark-induced and age-dependent leaf senescence by regulating the same set of target genes as PWR and HDA9.^54^ In this study, we discuss how HOS15 functions together with the PWR–HDA9 complex to promote histone deacetylation at the promoter regions of a common set of genes to suppress their expression, thereby regulating age-dependent and dark-induced senescence in Arabidopsis.

## HOS15 is an integral part of the PWR–HDA9 complex to control various traits

HOS15 interacts with PWR and HDA9 and functions within the same complex to co-regulate physiological processes and stress responses.^22^^,^^40^^,^^45^ For instance, HOS15 interacts with HDA9 and components of the evening complex LUX ARRHYTHMO (LUX), ELF3, and ELF4 to regulate the expression of GIGANTEA (GI) by modulating the histone acetylation level at its promoter, thereby controlling the flowering time.^28^ HOS15 interacts with PWR and HD2C to regulate the expression of cold-responsive (COR) genes by modulating the level of histone acetylation at their promoters, thus controlling the response to cold stress.^50^^,^^51^ In addition, PWR and HDA9 interact with each other as well as with ABSCISIC ACID INSENSITIVE 4 (ABI4) to regulate the expression of ABA catabolism genes (e.g., *CYP707As*) by modulating the level of histone acetylation at their promoter regions, thereby controlling ABA and drought stress responses.^23^^,^^25^ In a previous study, PWR and HDA9 were shown to regulate flowering time by suppressing the expression of *AGAMOUS-LIKE 19 (AGL19)* through the modulation of H3 acetylation.^55^ Interestingly, mutations in any individual component of the HOS15–PWR–HDA9 complex (*hos15–pwr–hda9)* led to its instability,^39^ thus highlighting the essential role of each component in maintaining the integrity of the complex. In addition, the HOS15–PWR–HDA9 complex regulates the expression of *AGL19* by modulating the level of histone acetylation at its promoter, thereby influencing floral transition.^26^ Furthermore, mutations in *HOS15, PWR,* or *HDA9* (*hos15, pwr,* or *hda9*, respectively) result in similar phenotypes, including early flowering, short plants, dwarfism, and short, blunted siliques.^26^ These findings indicate that the HOS15–PWR–HDA9 complex is a well-established regulatory module that controls various physiological processes and stress responses in a similar manner.

## HOS15 functions within the PWR–HDA9 complex to regulate senescence via common genes

**Figure 1.**
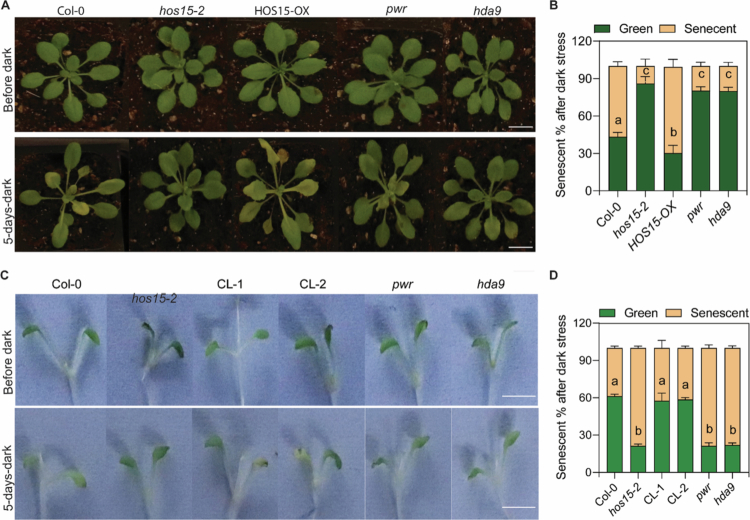
*hos15/pwr/hda9* mutants delay the dark-induced senescence phenotype. (A) Arial phenotypic comparison of plants before and after dark stress (scale bar = 2 cm). The seeds of the wild-type (Col-0), *hos15-2*, *35S:HOS15-GFP*, *pwr,* and *hda9* plants were grown on Murashige and Skoog (MS) medium for 12 d. The 12-day-old seedlings were transferred to the soil and covered with a transparent cover for 3 d. The 24-day-old plants were subjected to dark stress for 5 d by covering them with aluminum foil. The photographs were taken before and after dark stress. (B) Quantification of the percentage of senescent leaves (A) after dark stress is shown in the graph. (C) Phenotypic comparison of seedlings before and after dark stress (scale bar = 2 cm). The seeds of Col-0, *hos15-2*, two complemented lines (CL-1 and CL-2), *pwr,* and *hda9* plants were subsequently grown on MS media for 7  d. Seven-day-old seedlings were exposed to dark stress for 5 d by covering them with aluminum foil. The photographs were taken before and after dark stress. (D) Quantification of the percentage of senescent leaves (C) after dark stress is shown in the graph. Each experiment was repeated twice, and similar findings were obtained. Statistical differences were calculated using one-way ANOVA. The lowercase letters above each bar indicate statistically significant differences as determined by Tukey's test (*P* < 0.05).

**Figure 2.**
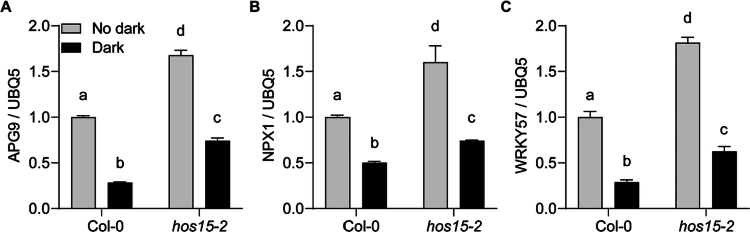
Transcript levels of *APG9*, *NPX1*, and *WRKY57* in dark-induced senescence leaves. (A–C) Comparative analysis of the *APG9*, *NPX1*, and *WRKY57* transcripts in dark-induced senescence leaves. Rosette leaves of the 4-week-old Col-0 and *hos15-2* plants were exposed to dark stress for 5 d. Total RNA was extracted from dark-treated and untreated leaves, and cDNA was produced. qRT-PCR was performed, and the expression levels of *APG9*, *NPX1*, and *WRKY57* were analyzed. UBQ5 was used as the normalization control. The experiment was repeated twice, with similar findings. The significant differences were calculated using two-way ANOVA. The lowercase letters above each bar indicate statistically significant differences as determined by Tukey's test (*P* < 0.05).

**Figure 3.**
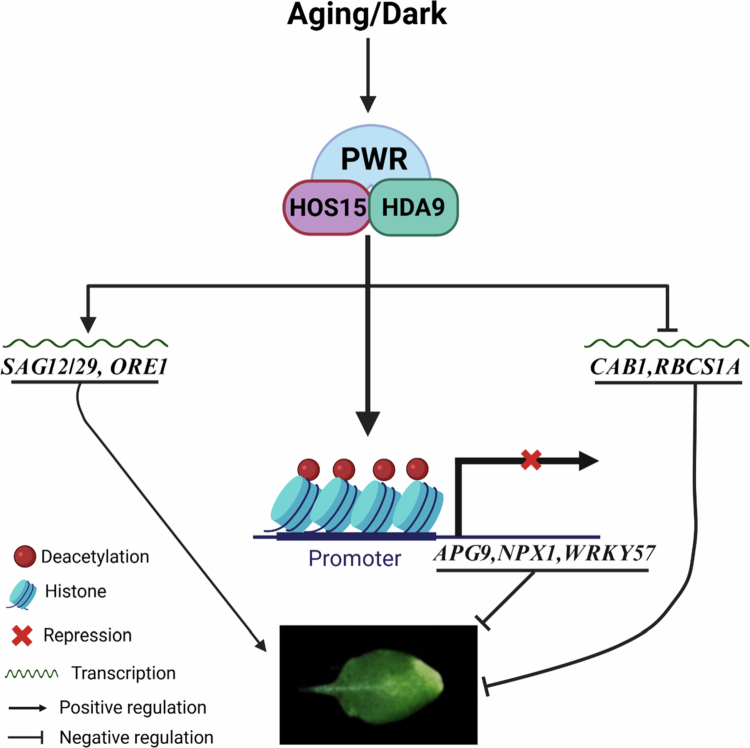
Working model of the HOS15–PWR–HDA9 complex in senescence. According to the proposed model, the HOS15–PWR–HDA9 complex functions as a coordinated regulatory unit that modulates gene expression during age-dependent and dark-induced senescence in Arabidopsis. Under these conditions, the complex promotes the expression of senescence-related genes, such as *SAG12, SAG29,* and *ORE1*, which function as positive regulators of senescence. In contrast, this complex also represses the expression of photosynthesis-related genes, such as *CAB1* and *RBCS1A*, which are considered to be regulators of senescence. In addition, the HOS15–PWR–HDA9 complex associates with the promoters of the *NPX1, APG9,* and *WRKY57* genes and suppresses their expression by promoting histone deacetylation at their loci. Despite the dual regulatory role of activating pro-senescence genes and repressing anti-senescence or photosynthesis-related genes, this complex orchestrates the progression of aging and dark-induced senescence in Arabidopsis.

Because HOS15, PWR, and HDA9 interact to form a corepressor complex, they collectively regulate the transcription of genes involved in key plant physiological processes.^15^^,^^40^^,^^56^ However, PWR and HDA9 function in distinct ways during ABA signaling and drought stress.^57^ The loss-of-function PWR and HDA9 mutants showed ABA-insensitive and drought-sensitive phenotypes.^23^^,^^25^ In contrast, HOS15 mutant (*hos15-2*) plants are tolerant to drought and ABA-sensitive.^48^ Moreover, PWR and HDA9 have been shown to regulate age-dependent and dark-induced senescence through a common set of target genes.^15^ However, whether HOS15, is involved in the regulation of leaf senescence has not yet been reported. Thus, in the recent past, we reported that HOS15 modulates age-dependent and dark-induced senescence in Arabidopsis.^54^ We showed that, similar to the *pwr* and *hda9* mutants, the loss-of-function HOS15 mutant plants exhibited delayed senescence through high chlorophyll content accumulation. In addition, the expression levels of senescence-related genes, such as *ORESARA 1 (ORE1) or NAC-domain transcription factor 092 (ANAC092)*, *SENESCENCE-ASSOCDIATED GENE 12 (SAG12)*, and *SENESCENCE-ASSOCDIATED GENE 29 (SAG29),* were lower in *hos15-2* plants compared to those of Col-0 plants. Whereas the transcript levels of photosynthesis-responsive genes, such as *CAB1* and *RBCSIA,* were higher in *hos15-2* plants than in the Col-0 plants.^54^ These findings suggest that HOS15 regulates leaf senescence by maintaining a relatively high chlorophyll content and modulating the expression levels of senescence- and photosynthesis-related genes. In addition to plant age, abiotic and biotic stresses also contribute to the progression of senescence.^3^ In this context, dark stress is crucial, empirically proven, and commonly used to induce senescence across plant species.^2^ The loss-of-function PWR and HDA9 mutants (*pwr* and *hda9*) delay the onset of the dark-induced senescence phenotype;^15^ therefore, we were interested in investigating whether HOS15 is also involved in dark-induced senescence. Importantly, we found that *hos15-2* plants exhibited delayed plant and seedling yellowing/senescence phenotypes similar to that of *pwr* and *hda9* mutants after the dark treatment compared to that of Col−0 plants (Figure 1A–D). The expression levels of senescence-related genes, such as *ORE1*, *SAG12*, and *SAG29,* were downregulated in *hos15-2, pwr,* and *hda9* mutant plants. In contrast, photosynthesis-related genes, such as *CAB1* and *RBCSIA,* were upregulated in *hos15-2, pwr,* and *hda9* mutant plants compared with those in Col-0 plants under dark stress,^54^ thus indicating that HOS15–PWR–HDA9 promotes dark-induced senescence via common target genes. Transcriptomic data from earlier studies showed that PWR and HDA9 regulate 90% of HOS15-controlled genes,^40^ which are involved in the response to different environmental stimuli. Genome-wide occupancy profiling data showed that HDA9 also associates with the promoters of important senescence negative regulator genes, such as *NPX1, APG9*, and *WRKY57.*^58^ PWR–HDA9 negatively regulates senescence via the same set of negative senescence regulators, such as *NPX1, APG9,* and *WRKY57,* by associating with their promoter regions and negatively regulating their expression levels.^15^ *WRKY57* regulates jasmonic acid (JA)-induced senescence by repressing the expression levels of key SAGs, including SAG12 and SENESCENCE4 (SIN4).^59^ Moreover, NUCLEAR PROTEIN 1 (NPX1) functions as a negative regulator of ABA signaling,^60^ and APG9 negatively regulates the expression of the SAGs such as SENESCENCE1 (SIN1) and YELLOW STRIPE LIKE4 (YSL4).^61^ Interestingly, we also reported that HOS15 negatively regulates the expression levels of negative senescence regulators, such as the *NPX1, APG9*, and *WRKY57* genes, through associating with their promoter regions.^54^ Therefore, because HOS15 also exhibits a delayed dark-induced leaf-yellowing phenotype (Figure 1), we tested whether HOS15 negatively regulates the expression levels of the senescence negative regulators *WRKY57, APG9,* and *NPX1* genes under dark-induced senescence. As expected, the expression levels of the senescence negative regulators *APG9, NPX1,* and *WRKY57* were upregulated in the *hos15-2* mutant plants compared to those in the Col-0 plants under both conditions (Figure 2A–C), suggesting that HOS15 negatively regulates the transcription levels of the senescence negative regulators *WRKY57, APG9*, and *NPX1* under dark-induced senescence, thus exhibiting similar roles to those of PWR and HDA9. In addition, HOS15, HDA9, and PWR play an upstream regulatory roles in several target genes to control various physiological processes and stress responses.^28^^,^^39^^,^^46^^,^^48^^,^^49^^,^^51^^,^^53^^,^^62^ Therefore, we examined the senescence phenotype of double and triple mutants of HOS15, PWR, and HDA9 and found that the *hos15-2, hos15pwr, hos15hda*9, and *hos15pwrhda9* mutants all presented delayed senescence similar to that of their respective single mutants,^54^ indicating that these genes function genetically in a parallel manner. Furthermore, the transcript levels of the senescence negative regulators *NPX1, APG9,* and *WRKY53* were increased in these (*hos15, pwr, hda9*) mutant plants compared to those in the Col-0 plants,^54^ which suggests that HOS15–PWR–HDA9 work together in the same complex to regulate senescence via common target genes (Figure 3).

## HOS15–PWR–HDA9 epigenetically controls senescence

Epigenetics is defined as heritable alterations in chromatin modification and gene expression without inducing any changes in the DNA code. DNA methylation, histone modification, chromatin remodeling, RNA methylation, and non-coding RNA regulation provide interrelated pathways to alter chromatin conformation and stabilize gene expression in a broad sense.^20^ Histone conformation is dynamically changed during cell senescence induced by age and environmental stimuli.^3^^,^^19^^,^^63^^,^^64^ Thus, dynamic chromatin reorganization is an important mechanism for controlling leaf senescence by altering the gene expression patterns. Several chromatin modifiers have been reported to be involved in plant leaf senescence.^19^ The JmjC domain-containing H3K4-specific demethylase JMJ16 suppresses H3 acetylation in age-dependent senescence via its demethylase activity, leading to the regulation of SAG genes.^65^ Recently, we reported that the HOS15–PWR–HDA9 complex regulates the expression levels of several genes by modulating their histone status, thereby regulating various physiological processes and stress responses.^22^ Since HDA9 lacks DNA-binding domain, it cannot directly recognize the target gene promoter, though, DNA-binding protein are required for specific sequence binding.^66^ PWR acts as a scaffold or adaptor protein, often interacting with sequence-specific transcription factors.^67^ PWR interacts with HDA9 and is essential for its nuclear localization and chromatin binding. Without PWR, HDA9 enrichment at the promoter of target genes is reduced, and HDA9 accumulation in the nucleus is also impaired.^15^ In addition, HDA9 binding peaks are located in promoter regions and are preferentially enriched in the promoters of active genes, but not in those of silent genes.^15^ Similarly, HDA9 is highly enriched in gene-rich euchromatic regions and depleted in repeat-rich centromeric heterochromatin. In addition, PWR is also enriched in the same regions as HDA9, and the binding of HDA9 to these loci requires PWR in vivo,^15^ suggesting that PWR recruits HDA9 to the nucleus and also binds to the promoters of target genes. PWR and HDA9 also promote senescence by regulating the H3 acetylation at the promoter regions of the target genes.^15^ We previously demonstrated that *hos15, pwr, hda9, hos15pwr, hos15hda9*, and *hos15–pwr–hda9* mutants increased the global H3 acetylation levels compared to WT plants,^54^ thus suggesting that the PWR–HDA9–HOS15 complex negatively regulates the levels of H3 acetylation. Furthermore, we have shown that the levels of H3 acetylation decrease with increasing leaf age; H3 status is substantially lower in late senescent leaves compared to those of young leaves.^54^ Furthermore, it was recently reported that high light conditions increased the H3 acetylation status.^68^ In contrast, we found that dark-induced senescence reduced H3 acetylation in WT plants while these levels were unaffected in *hos15-2* mutant plants as well as in the *hos15/pwr, hos15/hda9,* and *hos15/pwr/hda9* mutants;^54^ therefore, we suggest that the dark-induced H3 acetylation requires functional HOS15 and PWR–HDA9. As HOS15 functions in the same repressive PWR–HDA9 complex to control the H3 acetylation status of many genes.^22^ H3 acetylation levels in the promoter regions of the senescence negative regulators *NPX1, APG9,* and *WRKY57* genes were analyzed in *hos15-2* plants, which were substantially increased compared to those of WT plants. Taken together, these findings demonstrate that HOS15 works in the same repressive PWR–HDA9 complex and modulates aging and dark-induced senescence by controlling the expression of the same genes by modifying histone acetylation levels at their promoter regions in Arabidopsis.

## Summary

In response to numerous environmental signals and to pursue plant growth and development, the transcriptional co-repressor complex HOS15–HDA9–PWR modulates the acetylation status at the promoter of many genes.^40^^,^^56^ These three genes have been demonstrated to play a role in various stress signaling pathways, including freezing and drought stress, flowering transition, ABA signaling, response to pathogens, leaf size, and morphological features, either separately or in combination.^26^^,^^28^^,^^40^^,^^46^^,^^48^^,^^51^^,^^56^ Because the PWR–HDA9 complex interacts with *WRKY53*, PWR recruits HDA9 for the transcriptional suppression of the *NPX1, APG9,* and *WRKY57* genes to regulate age-dependent and dark-induced senescence.^15^ We found that HOS15 regulates the transcript levels and is directly associated with the *NPX1, APG9,* and *WRKY57* genes and that it modulates H3 acetylation at the promoter regions of these genes, thus promoting age-dependent and dark-induced senescence. According to our model, HOS15 forms a complex with HDA9 and PWR (HOS15-HDA9-PWR) to regulate senescence by inhibiting the transcript levels of photosynthesis-related genes, such as *CHLOROPHYLL A/B BINDING PROTEIN 1 (CAB1)* and *RUBISCO SMALL SUBUNIT 1A (RBCS1A)*. However, the increased transcript levels of senescence-related (senescence-positive regulators) genes, such as *ORE1*, *SAG12*, and *SAG29,* may be because they regulate senescence. Moreover, the HOS15–PWR–HDA9 complex inhibits the gene transcription of *NPX1*, *APG9*, and *WRKY57* because of its direct association with their promoter and promotes deacetylation at their promoter regions (Figure 3), thereby enhancing age-dependent and dark-induced senescence in Arabidopsis. Given the similar mechanistic role of the HOS15–PWR–HDA9 complex in regulating leaf senescence and flowering time in Arabidopsis, investigating the role of this complex in other physiological processes and stress responses would be interesting in the future. Further studies are needed to define the regulatory mechanisms of the HOS15–PWR–HDA9 complex that operate differently among plant species, particularly in response to leaf senescence.

## Data Availability

The data that support the findings of this study are available.
